# AAPM medical physics practice guideline 10.a.: Scope of practice for clinical medical physics

**DOI:** 10.1002/acm2.12469

**Published:** 2018-10-19

**Authors:** Jessica B. Clements, Christopher T. Baird, Steven F. de Boer, Lynne A. Fairobent, Tyler Fisher, James H. Goodwin, Dustin A. Gress, Jennifer Lynn Johnson, Kathryn L. Kolsky, Gig S. Mageras, Rebecca M. Marsh, Melissa C. Martin, Brent Parker, Daniel C. Pavord, Michael C. Schell, J. Anthony Seibert, Donna M. Stevens, Russell B. Tarver, Christopher G. Waite‐Jones, Nicholai Wingreen

**Affiliations:** ^1^ Southern California Permanente Medical Group Los Angeles CA USA; ^2^ Landauer Medical Physics Glenwood IL USA; ^3^ Department of Radiation Medicine Roswell Park Cancer Institute Buffalo NY USA; ^4^ Consultant Reston VA USA; ^5^ Therapy Physics, Inc. Signal Hill CA USA; ^6^ University of Vermont Medical Center Burlington VT USA; ^7^ Department of Imaging Physics The University of Texas MD Anderson Cancer Center Houston, TX USA; ^8^ Department of Radiation Physics The University of Texas MD Anderson Cancer Center Houston TX USA; ^9^ Mayo Clinic Rochester MN USA; ^10^ Department of Medical Physics Memorial Sloan‐Kettering Cancer Center New York NY USA; ^11^ Radiology School of Medicine University of Colorado Aurora CO USA; ^12^ Division of Physics and Engineering Department of Radiation Oncology The University of Texas Medical Branch Galveston TX USA; ^13^ Health Quest Poughkeepsie NY USA; ^14^ University of Rochester Medical Center Rochester NY USA; ^15^ Department of Radiology U.C. Davis Medical Center Sacramento CA USA; ^16^ Northwest Permanente, PC Clackamas OR USA; ^17^ The Center for Cancer and Blood Disorders Fort Worth TX USA; ^18^ Centennial Medical Physics Fort Collins CO USA; ^19^ AAPM Staff

## Abstract

The American Association of Physicists in Medicine (AAPM) is a nonprofit professional society whose primary purposes are to advance the science, education, and professional practice of medical physics. The AAPM has more than 8000 members and is the principal organization of medical physicists in the United States. The AAPM will periodically define new practice guidelines for medical physics practice to help advance the science of medical physics and to improve the quality of service to patients throughout the United States. Existing medical physics practice guidelines will be reviewed for the purpose of revision or renewal, as appropriate, on their fifth anniversary or sooner. Each medical physics practice guideline (MPPG) represents a policy statement by the AAPM, has undergone a thorough consensus process in which it has been subjected to extensive review, and requires the approval of the Professional Council. The medical physics practice guidelines recognize that the safe and effective use of diagnostic and therapeutic radiation requires specific training, skills, and techniques as described in each document. As the review of the previous version of AAPM Professional Policy (PP)‐17 (Scope of Practice) progressed, the writing group focused on one of the main goals: to have this document accepted by regulatory and accrediting bodies. After much discussion, it was decided that this goal would be better served through a MPPG. To further advance this goal, the text was updated to reflect the rationale and processes by which the activities in the scope of practice were identified and categorized. Lastly, the AAPM Professional Council believes that this document has benefitted from public comment which is part of the MPPG process but not the AAPM Professional Policy approval process. The following terms are used in the AAPM's MPPGs:

- Must and Must Not: Used to indicate that adherence to the recommendation is considered necessary to conform to this practice guideline.
- Should and Should Not: Used to indicate a prudent practice to which exceptions may occasionally be made in appropriate circumstances.

## 1 INTRODUCTION

Qualified Medical Physicists (QMPs, AAPM Professional Policy 1^1^) are members of multidisciplinary health care teams. As medical specialists, QMPs are dedicated to ensuring the safety of patients, medical staff, and the general public and improving quality of care to ensure accurate diagnosis and treatment of disease. These outcomes are achieved through numerous and varied activities, many of which are outlined in the Appendix: Scope of Practice Combined Task List. The skills required to perform these tasks are uniquely obtained through the scientific and clinical training of a medical physicist. The appendix organizes tasks in imaging and therapy medical physics by categories including administrative, clinical, educational, informatics, equipment performance evaluation (EPE), quality, and safety. Each task or activity indicates the AAPMs recommendation on the involvement of the QMP:

1. Activity must be performed by a QMP
2. Activity must be performed or supervised by a QMP
3. Activity should include a QMP

If the activity must be supervised by a QMP, the level of supervision is listed. Each level of supervision is defined in AAPM Professional Policy 18‐B^2^:

General — the procedure is performed under a Qualified Medical Physicist's overall direction and control. The QMP's presence is not required during the performance of the procedure, but must be available by phone to provide assistance and direction if needed. Under General Supervision, the training of the personnel who actually perform the procedure and the maintenance of the necessary equipment and supplies are the responsibility of the QMP.
Direct — a Qualified Medical Physicist must exercise General Supervision and be present in the facility and immediately available to furnish assistance and direction throughout the performance of the procedure. Direct Supervision does not require that the QMP must be present in the room when the procedure is being performed.
Personal — a Qualified Medical Physicist must exercise General Supervision and be present in the room during the performance of the procedure.

If there is an existing standard or guideline related to the task or activity, it is referenced.

Currently, the practice of clinical medical physics varies greatly depending on discipline and practice environment. While some activities are well‐defined by federal standards or regulations, most are mandated by individual states, with considerable variation in the required qualifications of those providing medical physics services and support. Other clinical medical physics activities are critical to providing quality patient care but are not defined by any local or federal standard or regulation. While medical physicists have an invaluable role in research and technology development, this document will only address the practice of medical physics in a clinical setting.

The scope of practice described here identifies the activities and responsibilities that a medical physicist can undertake based on the individual's training, qualifications, and demonstration of competence. In addition to describing what duties *must* be performed by a QMP, the scope of practice also defines what duties must be overseen by a QMP and those which a QMP could perform to bring additional value. The rationale for these distinctions is grounded in practice standards and guidelines, qualifications and training, and needs for patient, employee, and public safety. Ultimately the scope of practice defined here is the position of the AAPM, not a compilation of regulatory requirements or accreditation guidelines. It is also important to note that some states may have additional restrictions on the qualifications required to perform the tasks listed here.

This document describes the overall responsibilities and qualifications of a clinical medical physicist and includes a specific, but not exhaustive, list of clinical activities that are performed by medical physicists for four areas of practice: radiation oncology, diagnostic, nuclear medicine, and magnetic resonance imaging. As technology and patient care processes change this list must be modified. The descriptions in some cases are deliberately broad in order to anticipate changes in healthcare. While some skills are common to all medical physicists, some activities require the expertise of a medical physicist qualified in one of these specific subspecialties. According to AAPM Professional Policy 1,^1^ a QMP is qualified to practice on in the subfield(s) in which they are certified. This distinction is made for the activities listed in the Appendix.

## 2 INTENDED USERS

The contents of this document can aid a QMP when determining an overall scope of practice for the performance of medical physics activities. It can also be referenced by administrators, regulators, and accreditation bodies to identify what qualifications are necessary for individuals performing medical physics activities. In accordance with the rules of AAPM Medical Physics Practice Guidelines, this document will be revised at intervals not to exceed 5 yr. Any adoption of these recommendations by other organizations must be incorporated by reference, to reflect any updates made to this practice guideline.

## 3 DEFINITIONS

Medical Physics is a branch of physics associated with the practice of medicine. The term Medical Physics, as it is used here, includes the subspecialties of diagnostic medical physics, therapeutic medical physics, nuclear medical physics, and magnetic resonance imaging (MRI) physics.

Radiation includes both ionizing and nonionizing radiation such as electromagnetic radiation, particulate radiation, and ultrasound. These modalities, used for diagnostic or therapeutic purposes as prescribed by a properly qualified practitioner, are herein described as radiological procedures.

Quality Management consists of activities and programs designed to assure desired performance in the procedures and systems used in health care.

The Qualified Medical Physicist (QMP) is an individual who is competent to practice independently in one or more of the subfields of medical physics, and meets the criteria set forth in the Definition of a Qualified Medical Physicist (AAPM Professional Policy 1^1^). In addition, a QMP must hold a professional medical physics license where required and should uphold the AAPM Code of Ethics (AAPM Professional Policy 24^3^).

## 4 RESPONSIBILITIES

The essential role of the QMP is to assure the safe and effective use of radiation in medicine. The QMP performs or oversees the scientific and technical aspects of procedures necessary to achieve this objective. In the clinical setting, this involves the use of ionizing or nonionizing radiation in the delivery of radiation oncology treatments or performance of diagnostic medical imaging, nuclear medicine, or MRI examinations and procedures.

To perform these duties, the medical physicist often collaborates with physicians, other physicists, engineers, dosimetrists, radiation therapists, radiologic technologists, nurses, information technology professionals, and administrative staff. Successful integration as a member of the healthcare team requires that a QMP have comprehensive knowledge of many aspects of patient care, even if the specific task is typically performed by a non‐QMP team member. For example, a QMP working in radiation therapy should have in‐depth knowledge of patient setup techniques and treatment planning, even though those activities may be performed by a technologist and dosimetrist, respectively. In addition, the medical physicist must communicate with and provide education for healthcare professionals and patients. A QMP must be an integral part of the team that uses radiation therapy equipment, diagnostic imaging equipment, or radioactive materials in medicine. Typically, a QMP is employed by, or has a professional services contract with, a facility or healthcare provider. The responsibilities of the QMP must be recognized and supported by the administration and medical director of the facility.

The QMP's scope of practice categorizes medical physics activities into the following areas:

- Administrative
- Clinical services
- Education
- Informatics
- Equipment performance evaluation (EPE)
- Quality
- Safety

In order to effectively fulfill these responsibilities, the QMP may delegate certain tasks to other non‐QMP individuals. However, the QMP is responsible for ensuring that those individuals are adequately trained for such tasks. Furthermore, the QMP is responsible for overseeing the delegated work performed by those individuals and maintains ultimate responsibility for that work. Quality Assurance or performance testing completed by a supervised individual must be reviewed, approved, and signed by the QMP.

The QMP should also have control over physics‐related work that is routinely performed by clinical personnel, such as dosimetrists, therapists, and technologists. If they are not directly supervised by the QMP, the policies and procedures, methods of work and quality management programs that ensure the accuracy of their work should be established or at least reviewed by the QMP.

The value of a QMP comes from the possession of unique expertise, obtained through specialized training and demonstrated competence through certification, as described in AAPM Professional Policy 1.^1^ When considering the entire healthcare team previously mentioned, each member brings value from specific areas. Medical physicists have been trained extensively in the interactions, detection, biology, and safe use of radiation. Their deep understanding of the performance of radiation equipment and information systems brings value to clinical problem solving and the technology assessment process. They also are regulatory and accreditation experts that ensure high quality and safe patient care. A QMP should maintain that expertise through continuing education (AAPM Professional Policy 1^1^). As new developments arise, a QMP must obtain additional training before assuming responsibility for clinical implementation of these techniques.

In every case, the QMP adheres to the scope of practice defined herein and to the AAPM Code of Ethics (AAPM Professional Policy 24^3^) in order to support access to quality care for patients. It is recommended that a QMP adhere to these principles regardless of AAPM membership.

## 5 QUALIFICATIONS

The QMP has been defined previously by AAPM Professional Policy 1.^1^ Various states require either licensure or registration in order to practice Medical Physics. The requirements for professional licensure or registration vary by state, but they all include education and work experience requirements as well as continuing education requirements. Registration may be required for specific tasks such as providing medical physics services for mammography facilities or to provide a survey of a linear accelerator vault. Links to state regulations and information about medical physicist licensure or registration can be found on the AAPM Government and Regulatory Affairs Website.

Maintenance of certification and life‐long learning requirements are designed to ensure that the QMP maintain expertise within this rapidly evolving field. Technological advancements in medicine have rapidly brought developments from research centers into common practice. These developments bring great benefits to patients, but require the expertise of a QMP to ensure proper and safe use in the clinic.

## 6 CLINICAL ACTIVITIES

The level of supervision specified in this scope of practice indicates the minimum level of supervision recommended by the AAPM. The supervising QMP may require a more restrictive level of supervision, based on the qualifications of the individual being supervised and the nature of the activity being performed (AAPM Professional Policy 18‐B^2^) For example, MPPG 3.a describes the relationship between the supervising QMP and trainees. Some activities listed here may require additional training, beyond that which is common to all QMPs in that subspecialty. The QMP is responsible for ensuring the possession of appropriate expertise to perform these activities. For example, a Diagnostic QMP may receive special training to be able to perform a subset of tasks that traditionally fall under the scope of practice of a Nuclear Medicine QMP. Conversely, a Nuclear Medicine QMP may have specialized training in CT, as it is used in multimodality imaging (e.g., SPECT/CT, PET/CT). Similarly, Radiation Therapy QMPs may have specialized training to provide services for on‐board imaging devices and CT scanners used in simulation for radiation therapy treatment planning.

The scope of practice of the QMP may include, but is not limited to:

- Therapy
- Diagnostic
- Nuclear Medicine
- Magnetic Resonance Imaging
- Medical Health Physics activities related to individual sub‐specialties

## 7 CONCLUSIONS

The tasks listed here are meant to be used as general guidelines. Additional restrictions on qualifications required to perform these tasks may be defined by individual states; accrediting bodies; professional recommendations, standards, and guidelines; or facility policies and guidelines. Furthermore, states and other accreditation or regulatory agencies are likely to change over time. It is the responsibility of the QMP to keep apprised of any changes in regulations. These recommendations are not binding by any regulatory or accreditation body unless specified by that organization. The recommendations of this Practice Guideline must be considered in conjunction with an individual facility's policies and guidelines. It is advisable for a QMP to consider where the recommendations of this document differ with those of the QMP's facility and to abide by the more restrictive of the two.

## APPENDIX 1 SCOPE OF PRACTICE COMBINED TASK LIST

### 1.1

**Table:** Administrative Tasks — may be applicable to all subspecialties

Description of practice	Activity must be performed by a QMP	Activity must be performed or supervised by a QMP	Activity should include a QMP	Existing standards or guidelines related to the task. The position of the AAPM may be different than the referenced reports and standards.
Participates in operations management (e.g., establish client expectations, allocate personnel, ensure expectations are met)			x	1, 2
Participates in staffing and budget discussions and decisions that impact clinical medical physics services			x	1, 2
Participates in initial and ongoing facility planning (e.g., facility layout optimization, life cycle management of imaging equipment)			x	1, 2, 3
Consults on selection of new equipment prior to purchase, including review and comparison of equipment specifications and performance			x	3
Supervises medical physics staff, including physicists, medical physicist assistants, medical physics residents, and medical physics students in compliance with all relevant regulatory requirements and appropriate professional documents (e.g., AAPM reports)	x			1, 2, 4
Ensures that all local and national regulations and accreditation requirements as relating to medical physics are met and maintained	x			
Oversees quality assurance and quality control programs to meet local and national regulations, accreditation organization(s) standards, and national recommendations	x			10, 11, 12, 13, 14, 15, 16
Establishes training and competency requirements and monitors maintenance of competencies for clinical medical physics tasks	x			9
For activities that require supervision by a QMP, identifies the educational background and competencies required by those delegated to perform these duties	x			10, 11, 12, 13, 14, 15, 16
Provides institutional consultation on the development of clinical programs that utilize medical physics	x			
Participates in Coding/Billing (documentation)			x	
Provides technical oversight of personnel (including, but not limited to, radiation therapists, technologists, dosimetrists, and service engineers)	x			3
Serves as the radiation safety officer for the facility			x	17
Serves as a member of the institution's Radiation Safety Committee			x	17
Develops procedures for the initial and continuing evaluation of radiation protection equipment and procedures			x	18, 19
Acts as the facility's MR Safety Expert or MR Safety Officer			x	20
Develops an MRI safety program			x	10, 12

**Additional information may be found in the following resources**
1. AAPM Report 38: The Role of a Physicist in Radiation Oncology: The Role of a Physicist in Radiation Oncology
2. AAPM Report 301: An Updated Description of the Professional Practice of Diagnostic and Imaging Medical Physics
3. American College of Radiology (ACR) Guide to Medical Physics Practice
4. AAPM Medical Physics Practice Guideline 3.a: Levels of supervision for medical physicists in clinical training
5. AAPM Report 175: Acceptance Testing and Quality Control of Dental Imaging Equipment
6. AAPM Report 118: Parallel Imaging in MRI
7. AAPM Report 151: Ongoing Quality Control in Digital Radiography
8. AAPM Report 100: Acceptance Testing and Quality Assurance Procedures for Magnetic Resonance Imaging Facilities
9. A Report from the AAPM Subcommittee on Guidelines for Competency Evaluation for Clinical Medical Physicists in Radiation Oncology, JACMP 17(4) p3‐14
10. ACR Accreditation programs for imaging
11. ACR Accreditation program for radiation oncology
12. The Joint Commission (TJC) Diagnostic Imaging Standards (2015)
13. Intersocietal Accreditation Commission (IAC) Accreditation Programs for Imaging
14. RadSite Accreditation Program for Imaging
15. American Society for Radiation Oncology (ASTRO) Apex Accreditation program for radiation oncology
16. American College of Radiation Oncology (ACRO) Accreditation program for radiation oncology
17. AAPM Report 160: Radiation Safety Officer Qualifications for Medical Facilities
18. Nuclear Regulatory Commission (NRC) Part 20: Standards for Protection Against Radiation
19. International Atomic Energy Agency (IAEA) Report 39: Applying Radiation Safety Standards in Diagnostic Radiology and Interventional Procedures Using X Rays
20. American Board of Magnetic Resonance Safety (ABMRS) certification is available for MRSE and MRSO
21. ACR‐AAPM Technical Standard for the Performance of Radiation Oncology Physics for External Beam Therapy

**Table:**

Description of practice	Activity must be performed by a QMP	Activity must be performed or supervised by a QMP	Activity should include a QMP	Existing standards or guidelines related to the task. The position of the AAPM may be different than the referenced reports and standards
**Clinical Services Tasks — may be applicable to all QMP subspecialties**				
Develops procedures for initial acceptance testing and ongoing equipment testing (e.g., annual testing, postservice testing), including who performs the test, the frequency of testing, tolerance levels, and what to do if the test is out of tolerance	x			1, 2, 3
Ensures that measurement equipment is calibrated according to manufacturer recommendations and regulatory guidelines		x (general)		5, 6, 7, 8
Maintains appropriate documentation of all quality assurance and calibration results		x (general)		
Participates in research and development either individually or as part of a broader clinical team including support for clinical trials			x	3, 4
Participates in the development of products and procedures relevant to medical physics through collaboration with equipment manufacturers and Research and Development scientists			x	10
Participates in evaluation of emerging technologies and incorporating technology innovations into clinical practice	x			3
Reviews service activities (e.g., software updates) that may impact dose or image quality and determines if further medical physics follow‐up is required		x (general)		
Develops and oversees processes to authorize release of clinical equipment after service	x			
Evaluates technical and clinical physics issues related to patient care and determines if further medical physics follow‐up is required	x			
Communicates with and educates patients, including discussions of risk			x	
Consults with other healthcare professionals regarding patient radiation dose and associated risks	x			
Performs radiation dose estimates from diagnostic and nuclear medicine exams, including peak skin dose estimates, individual patient dose estimates, and fetal dose estimates, to be reported to the health care team and patient	x			11, 12, 13, 14, 15
Performs individual patient dosimetry for therapies involving radiopharmaceuticals	x			
Performs calculations to determine the release status of patients receiving treatment with radiopharmaceuticals			x	46
Participates in or oversees the safe use of radiopharmaceuticals or radionuclides during therapeutic procedures (e.g. Y‐90)			x	47
Provides consultation regarding patient safety in MRI, such as SAR considerations, prevention of patient burns, implanted devices, etc.			x	
Provides consultation regarding patient safety in ultrasound, such as thermal and mechanical index considerations			x	
Evaluates appropriate imaging protocols for diagnostic and interventional imaging and simulation and image‐guided radiotherapy	x			16, 17, 18, 19
Ensures the safe and appropriate implementation and use of imaging procedures and equipment as they pertain to diagnostic and interventional equipment and radiotherapy (simulation, treatment planning, and treatment delivery)	x			6, 22, 23, 24, 25, 26, 27, 36, 37, 38, 39, 40, 41, 42, 43, 44, 45
Participates in developing policies and procedures related to the appropriate therapeutic use of radiation	x			29, 30, 31
**Clinical Services Tasks — applicable to therapeutic medical physics QMP subspecialty**				
Approves radiation oncology technical procedures prior to clinical use	x			29, 30, 21
Works with the medical practitioner to develop the dosimetric component of treatment plans. Reviews radiation oncology dosimetry information noted in patient records	x			31, 32, 33, 34, 35
Provides written reports as needed to assure accurate and appropriate choice of dose delivery for radiation therapy	x			
Is involved with the development and delivery of special radiotherapy procedures		x (direct)		

**Additional information may be found in the following resources**
1. AAPM Report 38: The Role of a Physicist in Radiation Oncology
2. AAPM Report 42: The Role of the Clinical Medical Physicist in Diagnostic Radiology
3. ACR Guide to Medical Physics Practice
4. American Board of Radiology (ABR) Maintenance of Certification Requirements
5. AAPM Report 48: The Calibration and Use of Plane‐Parallel Ionization Chambers for Dosimetry of Electron Beams
6. AAPM Report 46: Comprehensive QA for Radiation Oncology
7. AAPM Report 59: Code of Practice for Brachytherapy Physics
8. AAPM Report 68: Permanent Prostate Seed Implant Brachytherapy
9. ASTRO Safety is No Accident
10. ASTRO Safety is No Accident (§3.6.0)
11. The Joint Commission Standards for Accreditation
12. ACR‐AAPM Technical Standard for Management of the Use of Radiation in Fluoroscopic Procedures
13. AAPM Report 96: The Measurement, Reporting, and Management of Radiation Dose in CT
14. AAPM Report 220: Use of Water Equivalent Diameter for Calculating Patient Size and Size‐Specific Dose Estimates (SSDE) in CT
15. AAPM Report 50: Fetal Dose from Radiotherapy with Photon Beams
16. AAPM Medical Physics Practice Guideline 1.a.: CT Protocol Management and Review practice Guideline
17. AAPM Medical Physics Practice Guideline 6.a: Performance Characteristics of Radiation Dose Index Monitoring
18. ACR‐AAPM Practice Parameter for Diagnostic Reference Levels and Achievable Doses in Medical X‐Ray Imaging
19. AAPM Report 116: An Exposure Indicator for Digital Radiography
20. AAPM Report 67: Protocol for Clinical Reference Dosimetry of High‐energy Photon and Electron Beams
21. AAPM Report 62: Quality Assurance for Clinical Radiotherapy Treatment Planning
22. AAPM Report 83: Quality Assurance for Computed‐Tomography Simulators and the Computed‐Tomography‐Simulation Process
23. AAPM Report 101: Stereotactic Body Radiation Therapy
24. AAPM Report 106: Accelerator Beam Data Commissioning Equipment and Procedures
25. AAPM Report 142: Quality Assurance of Medical Accelerators
26. AAPM Report 179: Quality Assurance for Image‐Guided Radiation Therapy Utilizing CT‐based Technologies
27. AAPM Medical Physics Practice Guideline 2.a: Commissioning and Quality Assurance of X‐ray–based Image‐Guided Radiotherapy Systems
28. AAPM Medical Physics Practice Guideline 5.a: Commissioning and QA of Treatment Planning Dose Calculations
29. AAPM Report 283: Application of Risk Analysis Methods to Radiation Therapy Quality Management
30. AAPM Medical Physics Practice Guideline 4.a: Development, Implementation, Use, and Maintenance of Safety Checklists
31. ASTRO White Paper: A Review of Safety, Quality Management and Practice Guidelines for High Dose‐Rate Brachytherapy (2014)
32. ACR–AAPM Technical Standard for the Performance of Radiation Oncology Physics for External Beam Therapy
33. ASTRO White Paper: Quality and Safety Considerations in Stereotactic Radiosurgery and Stereotactic Body Radiation Therapy (SRS/SBRT) (2011)
34. ACR‐ASTRO Practice Parameters for Radiation Oncology
35. American College of Surgeons Commission on Cancer Cancer Program Standards (2016)
36. AAPM Report 192: AAPM and GEC‐ESTRO Guidelines for Image‐Guided Robotic Brachytherapy
37. AAPM Report 154: Quality Assurance of U.S.‐Guided External Beam Radiotherapy for Prostate Cancer
38. AAPM Report 147: Quality Assurance for Nonradiographic Radiotherapy Localization and Positioning Systems
39. AAPM Report 128: Quality Assurance Tests for Prostate Brachytherapy Ultrasound Systems
40. AAPM Report 104: The Role of In‐Room kV X‐Ray Imaging for Patient Setup and Target Localization
41. ACR–ASTRO Practice Parameter for Image‐Guided Radiation Therapy (IGRT) CSC/BOC 2014
42. AAPM Report 24: Radiotherapy Portal Imaging Quality
43. AAPM Report 58: Clinical use of Electronic Portal Imaging
44. AAPM Report 75: The Management of Imaging Dose During Image‐Guided Radiotherapy
45. AAPM Report 91: The Management of Respiratory Motion in Radiation Oncology
46. National Council on Radiation Protection & Measurements (NCRP) Report 155: Management of Radionuclide Therapy Patients
47. AAPM Report 144: Recommendations of the American Association of Physicists in Medicine on dosimetry, imaging, and quality assurance procedures for 90Y microsphere brachytherapy in the treatment of hepatic malignancies

**Table:** Education Tasks — may be applicable to all QMP subspecialties

Description of practice	Activity must be performed by a QMP	Activity must be performed or supervised by a QMP	Activity should include a QMP	Existing standards or guidelines related to the task. The position of the AAPM may be different than the referenced reports and standards
Participates in clinical education and training programs as needed to provide appropriate clinical training and supervision required for students.			x	1
Provides MRI safety training to health care team members and emergency responders			x	
Provides formal and informal radiation physics training for all members of the care team necessary for safe and effective care of patients and employee safety			x	2

**Additional information may be found in the following resources**
1. AAPM Report 249: Essentials and Guidelines for Clinical Medical Physics Residency Training Programs
2. ACR Guide to Medical Physics Practice

**Table:** Informatics Tasks — may be applicable to all QMP subspecialties

Description of practice	Activity must be performed by a QMP	Activity must be performed or supervised by a QMP	Activity should include a QMP	Existing standards or guidelines related to the task. The position of the AAPM may be different than the referenced reports and standards
Participates in informatics technology resource management			x	1
Participates in developing policies and procedures for electronic medical information security and privacy			x	1, 2, 3, 4, 5
Develops and manages a quality assurance program for data transfer between clinical systems in radiation oncology	x			1, 6, 7

**Additional information may be found in the following resources**
1. AAPM Report 201: Information Technology Resource Management in Radiation Oncology
2. AAPM Report 30: E‐Mail and Academic Computer Networks
3. AAPM Report OR01: Information Transfer from Beam Data Acquisition Systems
4. CAMPEP Standards for Accreditation of Residency Educational Programs in Medical Physics
5. ACR‐AAPM‐SIIM Practice Parameter for Electronic Medical Information Privacy and Security
6. ASTRO Safety is No Accident
7. ACR‐ASTRO Practice Guideline for 3‐D External Beam Radiation Planning and Conformal Therapy

**Table:**

Description of practice	Activity must be performed by a QMP	Activity must be performed or supervised by a QMP	Activity should include a QMP	Existing standards or guidelines related to the task. The position of the AAPM may be different than the referenced reports and standards.
**Equipment Performance Evaluation (EPE) Tasks — applicable to diagnostic medical physics QMP subspecialty**				
Performs EPE for primary interpretation displays and modality displays, excluding displays used in mammography				1, 2, 3, 4, 5, 6, 7
*Acceptance, annual, posrepair, and continuous quality assurance**		x (general)		
Performs EPE for primary interpretation displays and modality displays used in mammography				1, 5, 6
*Acceptance, annual, postrepair*	x			
*Continuous quality assurance**		x (general)		
Performs EPE for dental x‐ray systems (Excluding dental CT)				8, 9
*Acceptance*	x			
*Annual, postrepair*		x (general)		
*Continuous quality assurance**		x (general)		
Performs EPE for general x‐ray systems, including computed radiography and digital radiography systems				8, 10, 11
*Acceptance*	x			
*Annual, postrepair*		x (general)		
*Continuous quality assurance**		x (general)		
Performs EPE for fluoroscopy systems including general fluoroscopy, mobile C‐arms, and interventional angiography systems				8, 12, 13, 14, 15
*Acceptance*	x			
*Annual, postrepair*		x (direct)		
*Continuous quality assurance**		x (general)		
Performs EPE for cone‐beam CT systems, including those used for dental imaging and as part of an interventional fluoroscopy system				2
*Acceptance*	x			
*Annual, postrepair*		x (direct)		
*Continuous quality assurance**		x (general)		
Performs EPE for mammography systems				1, 20, 21, 22
*Acceptance*	x			
*Annual, postrepair*	x			
*Continuous quality assurance**		x (general)		
Performs EPE for ultrasound systems				1, 2, 3, 4, 23, 24
*Acceptance*	x			
*Annual, postrepair*		x (direct)		
*Continuous quality assurance**		x (general)		
*Performs EPE for gamma cameras and SPECT systems*				1, 2, 3, 4, 30, 31
**Equipment Performance Evaluation (EPE) Tasks — applicable to nuclear medical physics QMP subspecialty**				
*Acceptance*	x			
*Annual, postrepair*		x (direct)		
*Continuous quality assurance**		x (general)		
Performs EPE for PET systems				1, 2, 3, 4, 32, 33
*Acceptance*	x			
*Annual, postrepair*		x (direct)		
*Continuous quality assurance**		x (general)		
Performs EPE for nonimaging nuclear medicine equipment (e.g., dose calibrators, uptake probes, well counters)				1, 2, 3, 4, 34
*Acceptance*		x (general)		
*Annual, postrepair*		x (general)		
*Continuous quality assurance**		x (general)		
**Equipment Performance Evaluation (EPE) Tasks — applicable to therapy medical physics QMP subspecialty**				
Performs EPE for equipment used for external beam therapy, brachytherapy, simulation, image guidance, treatment planning, radiation measurement, including associated computer systems, algorithms, data and output				35, 36, 37, 38, 39
*Acceptance*	x			
*Annual, postrepair*	x			
*Continuous quality assurance**		x (general)		
**Equipment Performance Evaluation (EPE) Tasks — applicable to diagnostic and therapy medical physics QMP subspecialties**				
Performs EPE for CT systems used only for radiation therapy simulations or as part of an image‐guided radiotherapy system, including CT‐on‐rails, fan‐beam megavoltage CT, and kilovoltage CT				16
*Acceptance*	x			
*Annual, postrepair*		x (direct)		
*Continuous quality assurance**		x (general)		
**Equipment Performance Evaluation (EPE) Tasks — applicable to diagnostic and nuclear medical physics QMP subspecialties**				
Performs EPE for diagnostic CT systems, including the CT portion of PET/CT scanners that are used to obtain a diagnostic CT scan				1, 2, 3, 4, 17, 18, 19
*Acceptance*	x			
*Annual, postrepair*		x (direct)		
*Continuous quality assurance**		x (general)		
**Equipment Performance Evaluation (EPE) Tasks — applicable to diagnostic, MRI, and therapy medical physics QMP subspecialties**				
Performs EPE for MRI systems, including systems used for radiation therapy treatment planning				1, 2, 3, 4, 25, 26, 27, 28, 29
*Acceptance*	x			
*Annual, postrepair*		x (direct)		
*Continuous quality assurance**		x (general)		

*Continuous quality assurance refers to periodic testing that is performed daily, weekly, or monthly, often by a technologist. The procedures and frequency of testing is often described by the manufacturer
**Additional information may be found in the following resources**
1. American College of Radiology Accreditation Programs for Imaging
2. Intersocietal Accreditation Commission Programs for Imaging
3. RadSite Accreditation Programs for Imaging
4. The Joint Commission Diagnostic Imaging Standards (2015)
5. ACR‐AAPM‐SIIM Technical Standard for Electronic Practice of Medical Imaging
6. AAPM Report OR03: Assessment of Display Performance for Medical Imaging Sytems
7. AAPM Report 196: Technical Note: Gray Tracking in Medical Color Displays — a report of Task Group 196
8. US Environmental Protection Agency Federal Guidance Report No. 14 Radiation Protection Guidance for Diagnostic and Interventional X‐Ray Procedures
9. AAPM Report 175: Acceptance Testing and Quality Control of Dental Imaging Equipment
10. ACR‐AAPM Technical Standard for Diagnostic Medical Physics Performance Monitoring of Radiographic Equipment
11. AAPM Report 151: Ongoing Quality Control in Digital Radiography
12. ACR‐AAPM Technical Standard for Diagnostic Medical Physics Performance Monitoring of Fluoroscopic Equipment
13. AAPM Report 15: Performance Evaluation and Quality Assurance in Digital Subtraction Angiography
14. AAPM Report 58: Managing the Use of Fluoroscopy in Medical Institutions
15. AAPM Report 125: Functionality and Operation of Fluoroscopic Automatic Brightness Control/Automatic Dose Rate Control Logic in Modern Cardiovascular and Interventional Angiography Systems
16. AAPM Report 179: Quality assurance for image‐guided radiation therapy utilizing CT‐based technologies
17. ACR‐AAPM Technical Standard for Diagnostic Medical Physics Performance Monitoring of Computed Tomography (CT) Equipment
18. AAPM Report 39: Specification and Acceptance Testing of Computed Tomography Scanners
19. AAPM Report 111: Comprehensive Methodology for the Evaluation of Radiation Dose in X‐Ray Computed Tomography
20. Mammography Quality Standards Act (MQSA) Act of 1998
21. AAPM Report 29: Equipment Requirements and Quality Control for Mammography
22. AAPM Report 223: Radiation Dosimetry in Digital Breast Tomosynthesis
23. ACR‐AAPM Technical Standard for Diagnostic Medical Physics Performance Monitoring of Real Time Ultrasound Equipment
24. AAPM Report 65: Real‐Time B‐Mode Ultrasound Quality Control Test Procedures
25. ACR‐AAPM Technical Standard for Diagnostic Medical Physics Performance Monitoring of Magnetic Resonance Imaging (MRI) Equipment
26. AAPM Report 34: Acceptance Testing of Magnetic Resonance Imaging Systems
27. AAPM Report 100: Acceptance Testing and Quality Assurance Procedures for Magnetic Resonance Imaging Facilities
28. AAPM Report 118: Parallel Imaging in MRI: Technology, Applications, and Quality Control
29. AAPM Report 77: Practical Aspects of Functional MRI
30. ACR‐AAPM Technical Standard for Medical Nuclear Physics Performance Monitoring of Gamma Cameras
31. ACR‐AAPM Technical Standard for Medical Physics Performance Monitoring of SPECT‐CT Imaging Equipment
32. ACR‐AAPM Technical Standard for Medical Nuclear Physics Performance Monitoring of PET Imaging Equipment
33. ACR‐AAPM Technical Standard for Medical Physics Performance Monitoring of PET/CT Imaging Equipment
34. AAPM Report 181: The Selection, Use, Calibration, and Quality Assurance of Radionuclide Calibrators Used in Nuclear Medicine
35. American College of Radiology Accreditation program for radiation oncology
36. ASTRO Apex Accreditation program for radiation oncology
37. ACRO Accreditation program for radiation oncology
38. American College of Surgeons Commission on Cancer Program Standards (2016)
39. ACR‐AAPM Technical Standard for the Performance of Radiation Oncology Physics for External Beam Therapy

**Table:** Quality Tasks — may be applicable to all QMP subspecialties

Description of practice	Activity must be performed by a QMP	Activity must be performed or supervised by a QMP	Activity should include a QMP	Existing standards or guidelines related to the task. The position of the AAPM may be different than the referenced reports and standards.
Ensures all medical physics tasks and duties are in compliance with all applicable regulations related to the use of ionizing and nonionizing radiation	x			
Is involved with the recommended action and patient health effects analysis from radiation medical events or near misses	x			1, 2
Participates in an ongoing peer‐to‐peer review program. This may be performed with another QMP within the practitioner's institution or an extramural QMP	x			3, 4, 5
Serves on institutional committees (e.g., Risk Management, Quality Assurance, and Professional Staff) as needed to provide relevant information related to medical physics			x	6, 7, 8, 9, 10
Consults in developing policies and procedures related to the appropriate clinical use of radiation for imaging purposes (e.g., advantages and disadvantages of various imaging techniques)			x	
Provides imaging protocol consultation with radiologists and other health care providers			x	11, 12, 13
Works with radiologists and technologists to optimize imaging protocols, including technical scan parameters and appropriate use of dose‐optimization features available on equipment (e.g., automatic tube current modulation, iterative reconstruction, pulsed fluoroscopy, etc.)	x			
Works with technologists and radiologists to establish reference levels for monitoring radiation dose in general radiography, fluoroscopy, and CT‐guided interventions		x (direct)		11, 12, 16
Works with technologists and radiologists to set dose alert levels for diagnostic imaging procedures		x (direct)		12
Analyzes dose indices of aggregate data to guide imaging optimization efforts			x	
Oversees or participates in evaluation, maintenance, and utilization of radiation dose index monitoring software		x (general)		
Quality Tasks — applicable to therapy medical physics QMP subspecialty				
Develops and manages a comprehensive Quality Management Program that monitors, evaluates, and optimizes radiation oncology processes	x			1, 14, 15

**Additional information may be found in the following resources**
1. ACR‐ASTRO Practice Guideline for 3‐D External Beam Radiation Planning and Conformal Therapy
2. ASTRO Safety is No Accident (§3.4.12, 4.1.4)
3. ASTRO Safety is No Accident (Sec. 3.4.8, 4.1.5)
4. ASTRO APEx Accreditation program for Radiation Oncology
5. AAPM Report 103: AAPM Task Group 103 Report on Peer Review in Clinical Radiation Oncology Physics
6. European Commission Report 181 General Guidelines on Risk Management in EBRT
7. AAPM Report 38: The Role of a Physicist in Radiation Oncology
8. ASTRO Safety Is No Accident (Chapter 3)
9. ACR Accreditation Program for Radiation Oncology
10. ACR Guide to Medical Physics Practice
11. NCRP Report 168: Radiation Dose Management for Fluoroscopically‐Guided Interventional Medical
12. NCRP Report 172: Reference Levels and Achievable Doses in Medical and Dental Imaging: Recommendations for the United States
13. NCRP Report 174: Preconception and Prenatal Radiation Exposure: Health Effects and Protective Guidance
14. AAPM Report 201: Information Technology Resource Management in Radiation Oncology
15. ASTRO Safety Is No Accident
16. The Joint Commission Diagnostic Imaging Standards (2015)
17. ACRO Accreditation Program for Radiation Oncology

**Table:**

Description of practice	Activity must be performed by a QMP	Activity must be performed or supervised by a QMP	Activity should include a QMP	Existing standards or guidelines related to the task. The position of the AAPM may be different than the referenced reports and standards.
**Safety Tasks — may be applicable to all QMP subspecialties**				
Plans and specifies thickness, material, and placement of shielding needed to protect patients, workers, the general public, and the environment from radiation produced incident to diagnosis or treatment in consultation with the architect and facility representatives	x			1, 2, 3, 4, 5, 6, 7, 8, 9, 10, 11
Verifies and documents that the required shielding was properly installed and that the shielding design goals were met	x			1, 2
Participates in radiation disaster planning and recovery			x	
Performs safety assessments (Failure Mode Effects Analysis, etc.) for process improvement			x	
Establishes and oversees radiation and/or MR safety programs to meet local and national regulations, accrediting organizations' standards, and national recommendations			x	13, 14
Provides radiation safety training to physicians, technologists, nurses, and other hospital staff			x	
Develops specifications for personnel and patient radiation protection equipment			x	15, 16
Oversees radiation protection, policies and procedures, regulatory compliance, accreditation requirements, and adherence to national recommendations			x	
Provides ongoing education and training to technologists and other personnel in the safe handling of radioactive materials			x	
Participates in personnel exposure monitoring			x	
**Safety Tasks — applicable to diagnostic and MRI medical physics QMP subspecialties**				
Provides guidance regarding controlled access to MRI areas			x	
Ensures the safety of the MRI environment			x	14
**Safety Tasks — applicable to nuclear and therapy medical physics QMP subspecialties**				
Develops and oversees processes for the proper receipt, handling, storage, and disposal of radioactive materials within the hospital			x	
Acts as an Authorized Medical Physicist for radioactive materials	x			
Manages and maintains hot labs used for storage and preparation of radioactive materials			x	
Develops facility procedures to address and manage responses to radioactive spills			x	

**Additional information may be found in the following resources**
1. NCRP Report 147: Structural Shielding Design for Medical X‐Ray Imaging Facilities
2. AAPM Report 108: PET and PET/CT Shielding Requirements
3. AAPM Report 42: The Role of the Clinical Medical Physicist in Diagnostic Radiology
4. AAPM Report 45: Management of Radiation Oncology Patients with Implanted Cardiac Pacemakers
5. AAPM Report 47: AAPM Code of Practice for Radiotherapy Accelerators
6. NCRP Report 151: Structural Shielding Design and Evaluation for Megavoltage X‐ and Gamma‐Ray Radiotherapy Facilities
7. AAPM Report 74: Quality Control in Diagnostic Radiology
8. NCRP Report 59: Operational Radiation Safety Program
9. NCRP Report 99: Quality Assurance for Diagnostic Imaging
10. NCRP Report 133: Radiation Protection for Procedures Performed Outside the Radiology Department
12. NCRP Report 151: Structural Shielding Design and Evaluation
13. AAPM Report 109: Code of Ethics
14. AAPM Report 160: Radiation Safety Officer Qualifications for Medical Facilities
15. ACR Guidance Document on MR Safe Practices: 2013
16. NRC Part 20: Standards for Protection Against Radiation
17. IAEA Report 39: Applying Radiation Safety Standards in Diagnostic Radiology and Interventional Procedures Using X Rays
